# Retroperitoneal fibrosis; a single-centre case experience with literature review

**DOI:** 10.1093/rap/rky050

**Published:** 2018-12-10

**Authors:** Saqib Adnan, Aicha Bouraoui, Sampi Mehta, Siwalik Banerjee, Shaifali Jain, Bhaskar Dasgupta

**Affiliations:** 1 Rheumatology Department; 2 Urology Department, Southend University Hospital, Westcliff-on-sea, Essex; 3 Rheumatology Department, University Hospital Coventry and Warwickshire, Coventry; 4 Radiology Department, Southend University Hospital, Westcliff-on-sea, Essex, UK

**Keywords:** retroperitoneal fibrosis, vasculitis, periaortitis, inflammation, biological therapies, PET

## Abstract

**Objective:**

We present 13 patients with retroperitoneal fibrosis, focusing on clinical features, radiological characteristics, treatments and their outcomes.

**Methods:**

Retrospective review of the medical records was performed of all retroperitoneal fibrosis patients diagnosed and treated in our department between 2012 and 2017.

**Results:**

Twelve patients were male, with a median age of 64 years. Eleven patients presented with abdominal pain or back pain or both. Aetiologies varied from idiopathic to malignancy and vasculitis. Twelve patients had PET scans. These showed ^18^F-fluorodeoxyglucose-avid retroperitoneal soft tissue around the abdominal aorta in the vast majority, with five scans also demonstrating localized or generalized uptake by the aorta. In all cases except one, glucocorticoids were applied as the first-line therapy. Further immunosuppressive therapy was required in 10 cases.

**Conclusion:**

Our patients were male and older in age compared with the existing literature. PET scans were very helpful in diagnosis of retroperitoneal fibrosis. Rituximab was found to be an effective treatment in six of our patients.

**Key messages**

- Back or abdominal pain in association with constitutional symptoms is a common presentation of retroperitoneal fibrosis.
- Retroperitoneal fibrosis may be related to large vessel vasculitis and can sometimes be limited to the abdominal aorta.
- PET-CT is the best imaging modality for evaluation and monitoring of RPF.

## Introduction

Retroperitoneal fibrosis (RPF), also known as Ormond’s disease, is a condition characterized by aberrant fibroinflammatory tissue developing in the retroperitoneum, usually around the infra-renal portion of the abdominal aorta (AA) and iliac vessels [1].

Retroperitoneal fibrosis is a rare condition, with limited data on its epidemiology. Studies estimate its incidence at ∼0.1–1.3 cases/100 000 persons per year and the prevalence at ∼1.4 cases/100 000 population [2, 3]. This disease commonly occurs between the ages of 40 and 60 years. A male preponderance of disease incidence is noted, with a male-to-female ratio of 2:1 to 3:1 [4].

Retroperitoneal fibrosis is an idiopathic condition in about two-thirds of cases; however, it may also be associated with other conditions, such as autoimmune diseases, atherosclerotic aortic disease, medications, malignancies, infections or radiotherapy [5]. IgG4-related disease is now increasingly recognized as a cause of previously categorized idiopathic RPF [6].

Cross-sectional imaging techniques, such as CT and MRI, are the most commonly used modalities in the diagnosis of RPF. PET-CT is emerging as a recognized tool in identifying the aetiology, in assessing steroid responsiveness and in monitoring this disease [7]. Here, we present a case series of 13 patients with RPF, with emphasis on the clinical presentation, varied aetiology, PET-CT findings, treatment and outcomes.

## Methods

A retrospective review of the medical records was performed of all patients seen in our rheumatology department and diagnosed with RPF between 2012 and 2017. Written informed consents were obtained from all the patients. Ethical consent was not required for this study.

Data collection included demographic characteristics, smoking history, clinical presentations, co-morbidities, laboratory/immunology results, radiological findings, management and outcomes of the various treatments used.

## Results

Thirteen cases were diagnosed with RPF in the period between 2012 and 2017 (Table 1). The median age at the diagnosis was 64 (range 49–77) years, and 12/13 (92%) patients were male. All patients were symptomatic at presentation. A majority of the patients reported back pain (62%) and abdominal pain (AP; 62%). Constitutional symptoms were reported in 76% of cases. The duration of symptoms varied from 6 weeks to 24 months.

**Table 1:** Demographic and clinical characteristics of the patients with retroperitoneal fibrosis

	Age (years)/ sex	Presenting symptoms	Duration of symptoms	Co-morbidities	Constitutional symptoms	Baseline Hb (g/l)	**Baseline CRP**^a^ (mg/l)	**Baseline sCr**^a^**(µmol/l)**	**Baseline IgG4**^a^**(g/l)**
1	60/male	Lower AP and BP	2 months	IHD, HTN	Low-grade fever, lethargy	123	110	120	Normal (1.18)
2	77/male	BP	18 months	H/o prostate carcinoma	Weight loss	87	48	Normal	Raised (4.24)
				AF, left ventricular systolic dysfunction (ejection fraction 40–45%)					
				HTN					
3	61/male	BP	8 months	Lupus nephritis	Weight loss, lethargy	91	46	200–400	Normal (0.94)
				Ulcerative colitis					
				Asthma					
4	68/male	Lower AP and bilateral flank pain	8 months	IHD	Weight loss, night sweats	127	57	151	Normal (0.85)
				Aortic stenosis					
				HTN					
				Asthma					
5	57/male	Lower BP and AP	7 months	Lynch syndrome	None	125	34	127	Raised (2.06)
				H/o rectal carcinoma and multiple squamous cell skin carcinomas					
6	46/male	Left lower AP	1.5 months (6 weeks)	Nil	Weight loss, anorexia	128	77	423	Normal (0.58)
7	72/male	Lower AP and bilateral flank pain	6 months	H/o nasopharyngeal and cutaneous lymphomas	Weight loss, lethargy, night sweats	135	64	Normal	Not done
				Peripheral neuropathy					
				AF and HTN					
8	75/male	Lower BP and lower left AP	3 months	DM	None	115	19	Normal	Normal (0.46)
				HTN					
				H/o CABG					
9	72/male	Lower AP	12 months	COPD, type 2 diabetes mellitus	Weight loss, anorexia	111	35	Normal	Normal (0.07)
				Cholangiocarcinoma					
10	56/male	Buttock and right leg pain on walking	6 months	Type 2 diabetes mellitus	None	141	13	Normal	Normal (0.20)
11	69/female	Right loin	4 months	Nil	Weight loss	130	48	91	Normal (0.52)
12	77/male	No pain	9 months	HTN	Anorexia	143	2	142	Raised (1.39)
13	49/male	AP	24 months	Nil	Weight loss, fevers, night sweats	132	52	Normal	Normal (0.72)

a Reference values: CRP: <5; IgG4: 0–1.3; sCr: 45–83.

AF: atrial fibrillation; AP: abdominal pain; BP: back pain; CABG: coronary artery bypass graft; COPD: chronic obstructive pulmonary disease; DM: diabetes mellitus; Hb: haemoglobin; H/o: history of; HTN: hypertension; IHD: ischaemic heart disease; sCr: serum creatinine.

The main co-morbidities included hypertension in 6/13 (46%), diabetes mellitus in 3/13 (23%), ischaemic heart disease in 3/13 (23%), and a history of previous malignancy in 3/13 (23%). Seven of the 13 (54%) patients had a positive history of smoking, and two of these were current smokers.

Twelve of the 13 (92%) patients had raised CRP, and 6/13 (46%) had acute kidney injury (AKI) at presentation. Six of 13 (46%) required ureteric stenting either unilaterally or bilaterally. Three of 13 (31%) had positive ANAs. In two patients, ANAs were positive in low titres of 1:80, with a speckled pattern in one case and a nucleolar pattern in the other case (Case 1). ENA and anti-dsDNA antibodies were negative in both these patients. The third patient had lupus nephritis. In this patient, ANA titres were high (1:640), with a speckled pattern. Further tests in this patient revealed a strongly positive anti-RNP/sm antibodies, with negative anti-dsDNA antibodies. Low complement levels were found in 2/13 (15%). Three of 13 (23%) patients had raised IgG4 levels.

**Fig. 1:**
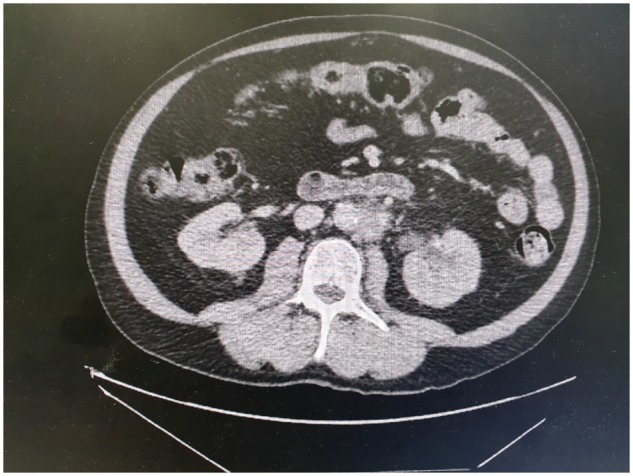
CT scan with contrast (Case 3), showing periaortic soft tissue

**Fig. 2:**
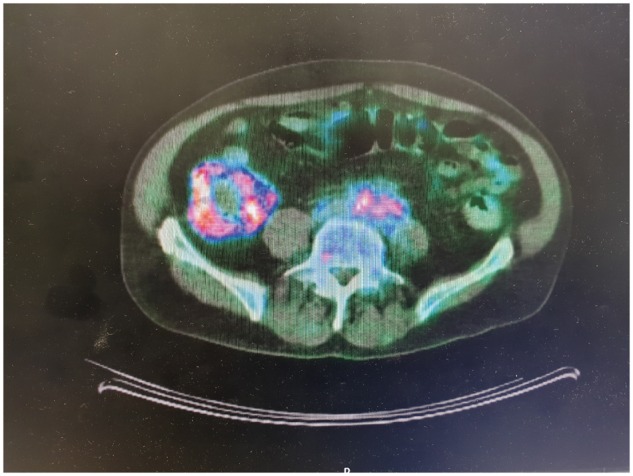
PET-CT scan of the same patient as in Fig. 1, demonstrating metabolic activity of the soft tissue

In all cases, the diagnosis of RPF was initially made or suspected on CT scan (Fig. 1). A PET scan was done in 11/13 (85%) cases, where the most common finding was an abnormal ^18^F-fluorodeoxyglucose (^18^F-FDG)-avid cuff of tissue around the AA (Fig. 2). Table 2 outlines the radiological findings in all cases.

**Table 2:** Radiological findings in patients with retroperitoneal fibrosis

	CT scan	MRI scan	PET scan
1	Possible osteomyelitis of L3/L4. Retroperitoneal inflammatory changes and fat stranding	MRI ruled out discitis	^18^F-FDG-avid periaortic soft tissue around the AA, with bilateral HN and hydro-ureter
2	Small periaortic lymphadenopathy and retroperitoneal soft tissue	Not done	Low-avidity tissue in the retroperitoneum. Some extension of this soft tissue along the common iliac vessels
3	Retroperitoneal mass and mild left HN	Not done	^18^F-FDG-avid soft tissue in retroperitonium with left-sided HN
4	Periaortic soft tissue around AA, with left-sided HN	MRI of small bowel showed evidence of RPF	Extensive uptake involving the aortic wall throughout its length. Abnormal ^18^F-FDG-avid cuff of tissue noted around AA
5	4.2 cm infra-renal AAA with periaortic stranding	Not done	Infra-renal aortic aneurysm, with ^18^F-FDG-avid soft tissue around it
6	Retroperitoneal soft tissue encasing AA and IVC. Bilateral HN	Not done	Not done
7	Infra-renal AAA, with enhancing soft tissue cuff extending along CIAs	Not done	Avid uptake in AAA extending to CIAs, with surrounding fat stranding
8	Periaortic soft tissue partly obstructing IVC	–	Patchy uptake in ascending and descending aorta
9	Abnormal cuff of soft tissue around AA	Not done	Large avid mass encircling AA, with underlying aneurysm
10	Cuff of soft tissue around infra-renal AA, right CIA and left internal iliac artery. High-grade stenosis of right internal iliac artery	Not done	Avid uptake in infra-renal AA, both common and internal iliac arteries
11	Right HN, with RPF at aortic bifurcation. Small right kidney	Not done	Not done
12	Right HN. Cuff of soft tissue around infra-renal AA and both CIAs	Not done	Moderately avid soft tissue extending around AA, extending along CIA. Patchy uptake by aortic wall
13	Retroperitoneal and pancreatic homogeneous mass, with right HN	Not done	Moderately avid, extensive soft tissue in retroperitonium, encasing pancreas

AA: abdominal aorta; AAA: abdominal aortic aneurysm; CIA: common iliac artery; ^18^F-FDG: ^18^F-fluorodeoxyglucose; HN: hydronephrosis; IVC: inferior vena cava; RPF: retroperitoneal fibrosis.

Three of the 13 (23%) patients had a biopsy of the retroperitoneal tissue, showing lymphocytic infiltrates with reactive changes in two cases and florid eosinophilic infiltration in one case. There was no evidence of lymphoma, metastatic carcinoma, granulomatous disease or IgG4-related disease in any of these cases.

The final diagnosis was idiopathic RPF in 4/13 (31%), and RPF secondary to large vessel vasculitis (LVV) in 5/13 (38%), which was localized to AA in 3/13 (23%). Two patients had an abdominal aortic aneurysm with periaortitis. One patient turned out to have lymphoplasmacytic lymphoma, and one was diagnosed with SLE and class 4 lupus nephritis. One patient with aortitis also had an abdominal aortic aneurysm. Table 3 describes the diagnoses, treatments and their outcomes.

**Table 3:** Diagnosis, treatment and outcomes of patients presenting with retroperitoneal fibrosis

	Aetiology	Steroid used	DMRDs/ biologics/ others	Clinical response	Biochemical response	Radiological response	Requirement for stents
1	Idiopathic (periaortitis)	i.v. MP followed by prednisolone	RTX	CR	(CR)	NA	Yes (bilateral) removed
					CRP 1		
					sCr 93		
2	LPL	Yes	Chemotherapy	CR	(CR)	CT (PR)	None
			(included RTX)		ESR 2	Significant resolution of mesenteric stranding	
					CRP 11		
					SIF N		
3	SLE	i.v. MP followed by prednisolone	CYC followed by MMF	CR	(PR)	PET-CT (CR)	Yes (left) still *in situ*
					CRP N	Remarkable improvement	
					C3/C4 N		
					sCr149		
4	LVV	i.v. MP followed by prednisolone	RTX	CR^a^	(PR)	PET-CT (CR) Metabolically inactive vessels	Yes (left) removed
					CRP 1		
					sCr 137		
5	AAA with periaortitis	i.v. MP followed by prednisolone	RTX	CR	(PR)	CT (PR)	No
					CRP 11	Slight improvement in inflammatory changes around AAA	
					sCr 94		
6	Idiopathic	i.v. MP followed by prednisolone	MMF	CR	(CR)	CT (PR)	Yes (bilateral) removed
					CRP 3	Slight reduction in pre- and para-aortic soft tissue	
					sCr 99		
7	Abdominal aortitis with AAA	Prednisolone	AZA	CR	(CR)	Non-contrast CT (CR)	No
					CRP 8	Only AAA	
8	LVV	None	No treatment	CR	(CR)	NA (spontaneous clinical remission)	No
					CRP 2		
9	AAA with periaortitis	i.v. MP followed by prednisolone	RTX	CR^b^	(CR)	PET-CT (CR)	No
					CRP 4	Resolution of periaortitis	
10	Abdominal aortitis	Prednisolone	RTX	CR	(CR)	PET-CT (CR)	No
					CRP 3	Complete resolution of periaortic inflammation	
11	Idiopathic	Prednisolone	None	CR	(CR)	CT (CR)	Yes (right) removed
					CRP 8	Resolution of hydronephrosis and RPF	
					sCr 99		
12	Abdominal aortitis	Prednisolone	None	CR	(PR)	CT (CR)	Yes (right) removed
					CRP 4	No sizeable RPF	
					sCr149		
13	Idiopathic	Prednisolone	MMF	CR	(PR)	CT (PR)	No
					CRP 14	Slight improvement in size of retroperitoneal soft tissue	

a Treatment complicated by recurrent respiratory infections and hypogammaglobulinaemia, probably RTX related.

b Developed cholangiocarcinoma 3 years later.

AAA: abdominal aortic aneurysm; CR: complete response; CRP: CRP (mg/l); LPL: lymphoplasmacytic lymphoma; LVV: large vessel vasculitis; MP: methylprednisolone; N: normal; NA: not assessed; RPF: retroperitoneal fibrosis; PR: partial response; RTX: rituximab; sCr: serum creatinine (µmol/l); SIF: serum immunofixation.

All our patients were treated with glucocorticoids (GCs) except one, in whom the disease was self-limited and who went into remission without any treatment. Six of the 13 (46%) patients required i.v. methylprednisolone followed by oral GCs. British Society of Rheumatology Giant Cell Arteritis guidelines were followed regarding the tapering of GCs. Initial doses of GCs were continued for at least 2–4 weeks before tapering by 10 mg every 2 weeks down to 20 mg prednisolone daily. The dose was then reduced further by 2.5 mg every 2 weeks down to 10 mg prednisolone daily. Afterwards, GCs were tapered very slowly until a 5 mg dose was reached. The aim was to maintain the patients long term on low-dose prednisolone ≤ 5 mg daily.

Four of the 13 (31%) patients required conventional immunosuppressive therapy (MMF in 3 and AZA in 1 case). Six of 13 (46%) were treated with rituximab (RTX), including one patient with lymphoplasmacytic lymphoma. The vast majority of patients had a good response to treatment, with normalization of CRP in 11/13 (85%). The AKI resolved completely in three, whereas three patients developed chronic kidney disease. In one case, this was attributable to class 4 glomerulonephritis related to SLE. One of the patients died from biliary sepsis and cholangiocarcinoma, which was diagnosed 3 years after the diagnosis of RPF. Another patient with Lynch syndrome died from an upper gastrointestinal bleed after developing duodenal adenocarcinoma.

## Discussion

Retroperitoneal fibrosis is a rare inflammatory disease with poorly understood aetiopathogenesis and is characterized by non-specific clinical signs. In this study, we describe 13 cases diagnosed with RPF. Our data show an overwhelmingly male predominance, 12:1 male/female ratio, compared with the literature (2:1 to 3:1) [4]. Furthermore, an increased proportion of our patients were older compared with those described in the literature (average age 67 *vs* 50–60 years) [8]. By and large, our patients had either AP or backache or both. The pain was constant day and night, severe infiltrative in character, with a normal spinal examination. Constitutional symptoms were noted in all except one, who only had raised inflammatory markers. At the time of presentation, 46% of patients had AKI.

These non-specific RPF symptoms were reported in the literature. The most common presentation is a chronic AP or backache associated with constitutional symptoms [9]. Of our patients, 62% had backache, which suggests that this is a relatively common presentation of inflammatory aortic disease and, when present with constitutional symptoms, should always arouse the possibility of this condition. Bilateral ureteral obstruction with AKI is very common, with figures ranging from 42 to 95% in different studies [5]. In one series, >60% of patients developed renal failure [6]. Some patients may also develop lower extremity oedema, scrotal swelling or constipation. Inflammatory markers are elevated in >50% of patients [5].

^18^F-Fluorodeoxyglucose-PET, which was used in 11 of our cases, has been recognized increasingly as a very effective modality, not only for the diagnosis of RPF but also for assessing its extension and the vascular and perivascular lesions. This imaging modality may reveal active vasculitis elsewhere and can disclose other areas of involvement in cases of malignancies and IgG4-related RPF [10–13]. ^18^F-Fluorodeoxyglucose-PET has also shown usefulness in predicting the response to GCs. In one study, the degree of ^18^F-FDG avidity was very well correlated with the responsiveness to GCs. In that study, patients with a negative PET scan had no response. Only a few patients with low-grade avidity had a measurable response. In contrast, a majority of patients with high-grade avidity showed a response to GCs [14]. PET may also be a useful tool during follow-up to assess the response to treatment and to detect disease relapse [7].

Five of our 11 patients who underwent PET scan also showed the presence of ^18^F-FDG avidity in the infra-renal AA, with or without extension along the iliac arteries and other segments of aorta. One of these patients had an abdominal aortic aneurysm. We suggest that these patients might have LVV, which in three cases was limited to the infra-renal aorta and its branches particularly, with raised inflammatory markers noted in four of these patients.

CT and MRI scans are important tools in the diagnosis of RPF. Typically, they show a soft tissue mass encasing the AA and common iliac arteries and often the ureters, leading to hydronephrosis [7]. These findings were also seen in our study. CT and MRI have been seen to lack the ability definitively to exclude malignancy as a cause of RPF. The degree of contrast enhancement on these imaging modalities may not accurately reflect the real metabolic activity in the area of involvement; hence, it may not reliably predict the response to steroid treatment [15].

As highlighted in our case series, RPF has a wide spectrum of aetiologies, varying from connective tissue disease, such as SLE, to lymphoma, vasculitis and idiopathic. There are many theories about the aetiopathogenenesis of idiopathic RPF. Previously, it was thought that this condition occurs as a complication of aortic atherosclerosis, which can explain the pathogenesis of RPF in cases of degenerative aortic disease. However, it fails to capture the majority of patients with RPF, many of whom have a complex systemic nature of their disease, with constitutional features, raised inflammatory markers and concomitant autoimmune diseases.

From our experience, it seems that LVV accounts for a significant proportion of idiopathic RPF. The condition may start as a primary aortitis, with the aortic inflammation triggering a fibroinflammatory response in the retroperitoneum. Indeed, in two of our patients the vessel wall inflammation was not limited to the infra-renal aorta and common iliac arteries but also involved the thoracic aorta and its branches. In a case series of seven patients with chronic periaortitis and RPF, PET scans demonstrated vascular uptake in the thoracic aorta and/or its branches in three (43%) patients [16].

Retroperitoneal fibrosis may occur in association with other autoimmune diseases. One of our cases with lupus nephritis presented with RPF. It is therefore essential that the patient undergoes investigations such as ANA, anti-CCP antibodies, RF, ANCA, C3 and C4 levels, urine microscopy and urine protein:creatinine ratio. Owing to the increased incidence of Hashimoto thyroiditis, thyroid function tests and thyroid peroxidase antibodies should also be checked in patients with idiopathic RPF [17].

Retroperitoneal fibrosis might be a manifestation of underlying malignancy (we had a case of lymphoplasmacytic lymphoma that presented with hypocomplementaemia and back pain). Various reports show that malignancy accounts for 8% of RPF cases [18]. In one study of 204 patients with RPF, the frequency of malignancy was 5.4% [19]. It is therefore important to exclude this carefully in the evaluation of the patient. If there are any concerns based on clinical or radiographic features, a retroperitoneal biopsy is warranted [20].

Management of RPF includes medical therapy and ureteral stenting in the event of severe hydronephrosis. Glucocorticoids are the mainstay of RPF treatment [21–23]. All our patients required GCs, except one case who had spontaneous resolution without any treatment. Glucocorticoids are very effective in inducing remission. In our series, all patients responded to prednisolone; however, the response was incomplete in five. This group included one patient with LVV, who was treated with RTX with a very good clinical, biochemical and radiological response.

We used high-dose GCs in patients with more severe presentations, such as significant renal impairment, or in those with extensive uptake on PET scan, especially by the aorta and/or its branches. High-dose GCs consisted initially of up to three pulses of 1 g i.v. methylprednisolone followed by 60 mg prednisolone daily. We used medium-dose prednisolone, in the range of 30–40 mg daily, in those with less severe disease. Unfortunately, a significant proportion of patients relapse after initial remission. In our series, several patients relapsed when the dosage of prednisolone was reduced to 5–10 mg daily. These patients were re-treated with a higher dose of prednisolone with gradual tapering, depending on the nature and intensity of relapse. These patients also required additional immunosuppressive therapy. This was in the form of RTX in those with more severe disease, as mentioned above, with good disease response. Three patients, including one with lupus nephritis, were treated with MMF. One patient received AZA for 3 months, which was later stopped, and patient remained in remission on low-dose prednisolone.

In one study, 16 relapsing patients were treated effectively to maintain remission with high-dose prednisolone tapered over a 12-month period along with MTX at a dose of 15–20 mg per week. This combination maintained remission in 79% of patients [24]. Another small case series showed the efficacy of AZA in RPF [25, 26]. There are some data on the efficacy of RTX and tocilizumab; however, there are no randomized controlled trials to assess the effectiveness of these treatments in RPF. Rituximab has been found to be very effective in RPF, especially in the context of IgG4-related disease [27].

Other immunosuppressive medications have also been used, such as MMF, CYC and CSA [6, 28, 29]. For its potential anti-fibrotic properties, tamoxifen has also been used in the treatment of RPF. However, it has been found to be less effective compared with GCs, both in terms of initial remission and in preventing future relapses [30].

Our study has some limitations. Firstly, it was a retrospective study. Secondly, the number of patients in the study was small, owing to which recommendations regarding the management of this condition could not be made. Thirdly, most of our patients did not have a retroperitoneal biopsy because it is an invasive procedure and requires specific expertise. We could therefore not comment on the possibility of IgG4-related RPF in many of our cases.

### Conclusion

We present a case series of RPF, emphasizing its presentation with back and AP and constitutional symptoms, and the need for early diagnosis and treatment to prevent irreversible renal damage. We highlighted the clinical and radiological (particularly ^18^F-FDG-PET) characteristics and the outcomes of medical management, including RTX. We demonstrated the role of PET scans in diagnosis and monitoring of patients with RPF. Large prospective studies and an RPF registry are required to understand the pathophysiology of this condition and to establish recommendations for its management.

*Funding*: This work received no funding from any source.

*Disclosure statement*: The authors declare no conflicts of interest.
